# Dispositional Self-Construal Modulates the Empathy for Others’ Pain: An ERP Study

**DOI:** 10.3389/fpsyg.2020.508141

**Published:** 2020-10-06

**Authors:** Jie Chen, Bijia Chang, Wenjie Li, Yupeng Shi, Haizhou Shen, Rong Wang, Lei Liu

**Affiliations:** ^1^School of Educational Science, Hunan Normal University, Changsha, China; ^2^Cognition and Human Behavior Key Laboratory of Hunan Province, Changsha, China; ^3^School of Psychological and Cognitive Science, Peking University, Beijing, China

**Keywords:** empathy, ERP, P3, dispositional self-construal, pain

## Abstract

Previous studies have shown that temporal self-construal priming can modulate the empathic neural responses to others’ pain. However, little is known about the influences of the dispositional self-construal on empathic neural responses to others’ pain. The present study aimed to investigate neural correlates that underlie the modulation effect of dispositional self-construal on perception of others’ pain. Event-related potentials were recorded for pictures depicting the hands of strangers in painful or no-painful situations while subjects performed a pain judgment task. The regression analysis on behavioral data showed that the level of interdependent self-construal could positively predict behavioral ratings of perceived pain, but not the self-unpleasantness. The ERP results showed painful stimuli elicited decreased N2 amplitudes and larger P3 amplitudes than those by no-painful stimuli. Moreover, the level of interdependent self-construal (interdependence minus independence scores) could predict the amplitude differences on the P3 component (painful minus neutral stimulus conditions), but not the N2 component: the higher the level of the interdependent self-construal, the larger amplitude differences of P3 to painful stimuli (vs. no-painful stimuli). These findings extended previous studies by showing a clear modulation effect of the dispositional self-construal on empathic neural responses to others’ pain, and that this modulation effect occurred at the late cognitive evaluation stage indexed by the P3 component.

## Introduction

Pain empathy refers to the capacity to understand and share others’ pain, as well as the corresponding affective-motivational reactions, which plays an important role in social communication and human interactions (Decety and Jackson, 2004). Previous neurocognitive studies have found that empathic neural responses to others’ pain could be modulated by both the dispositional and situational factors. The dispositional factors mainly refer to the empathizer’s inherent and stable characteristics such as personality characteristics, gender, and age (Han et al., 2008; Chiao et al., 2009; Mella et al., 2012), whereas the situational factors refer to the context in which the empathy takes place, such as the characteristics of the observed target who is in pain or the relationship between the target and the empathizer (Fan and Han, 2008; Cui et al., 2016). For example, it has been found that the level of social dominance orientation (SDO), a personality trait, had a significant negative correlation with responses to perceived pain in others within the left anterior insula and anterior cingulate cortex that are related to empathy for others’ pain (Chiao et al., 2009). An event-related potential (ERP) study showed that larger N2 amplitudes were elicited by painful than no-painful stimuli when the observed target was a moral or neutral one, whereas no such effect was found for the immoral one. Moreover, empathic neural responses to perceived pain were stronger to members of ingroup than to those of outgroup (Xu et al., 2009; Mathur et al., 2010; Sessa et al., 2014a).

Taken together, these findings indicate that empathic neural responses are modulated not only by what the pain-perceiver and target person are but also by the relationship between pain-perceiver and target person. Nevertheless, the relationship between the pain-perceiver and the target person is not static and actually depends on how the perceiver views and values this relationship. Self-construal is a construct, which refers to how people view themselves and their relation to others and the social environment (Cross et al., 2011). According to Markus and Kitayama (1991), people from individualistic cultures tend to perceive themselves as autonomous and independent from others (an independent self-construal), making them likely focus on uniqueness and self-interest; in contrast, people from collectivistic cultures define themselves as connected and interdependent with others (an interdependent self-construal), making them likely emphasize social harmony and others’ feeling and attitudes (Markus and Kitayama, 1991). In addition, although collectivist culture emphasizes harmony and cooperation, it has been found that people in collectivist culture are more alert to group members than those in individualistic culture (Liu et al., 2019). Moreover, it has been suggested that both the independent and interdependent self-construals exist in all cultures (Singelis, 1994; Oyserman et al., 2002; Sui et al., 2013). The present study aimed to investigate whether and how self-construal modulated neural correlates of pain perception in people from East Asian culture.

Previous ERP and neuroimage studies have shown that temporary shifts in self-construal can modulate the neural activities underlying various cognitive processes such as visual attention, pain perception, self-other processing, and motor-related processing (Sui and Han, 2007; Lin and Han, 2009; Harada et al., 2010; Obhi et al., 2011; Sui et al., 2013; Wang et al., 2014). More recently, Jiang et al. (2014) used ERPs to investigate the influences of self-construal priming on empathic neural responses to others’ pain both in the Chinese and Westerners and found that the amplitude differences to painful pictures (vs. no-painful pictures) were decreased under interdependent self-construal priming among the Chinese and under independent self-construal priming among Westerners during 232–332 ms (Jiang et al., 2014). A recent fMRI study showed that the interdependent self-construal priming increased neural activities in the mid-cingulate cortex (MCC), left insula, and supplementary motor area (SMA) when viewing racial ingroup compared with out-group members in pain. In contrast, the racial in-group bias in neural responses to others’ pain in the MCC, left insula, and SMA was eliminated by priming independent self-construal (Wang et al., 2015).

These studies suggested that empathic neural responses to others’ pain could be modulated by temporary activation of self-construal through a situational prime. However, unlike the temporary situational self-construal, the dispositional self-construal is a stable trait, the formation of which is influenced by long-term cultural experiences (Sui et al., 2013). Moreover, it was suggested that the self-construal priming tasks might induce or activate other dimensions of culture that are unrelated to self-construal (Cross et al., 2011). Thus, the present study directly measured the dispositional self-construal by the self-construal scale (SCS; Singelis, 1994) and investigated its relation to neural activity underlying the perception of others’ pain using ERPs.

It has been found that the ERP waves elicited by painful vs. no-painful stimulation diverged from the early stage (the N1 and P2 components) to the later stage (the N2 and P3 components) (Fan and Han, 2008; Decety et al., 2010; Sessa et al., 2014a,b). The N1 and P2 responses to perceived pain reflect an early automatic process of empathy for pain (Fan and Han, 2008; Decety et al., 2010), while the P3 component is known to reveal a late, top-down controlled process involved in cognitive evaluation of pain. The N2 component, a temporal stage where information processing occurs between automatic and controlled phases, is thought to reflect the attention-orienting response to emotionally salient stimuli (Nagy et al., 2003; Carretié et al., 2004). It has been found that temporary situational self-construal could modulate the intermediate component of empathy indexed by the N2 component rather than the later controlled component of empathy indexed by the P3 component (Jiang et al., 2014). The present study further aimed to determine whether the modulation effects of dispositional self-construal on neural empathic responses to perceived pain occurred on the early automatic empathic stage, the later attention orienting stage, or the late cognitive evaluation stage. We expected that the levels of dispositional self-construal would be correlated with average amplitudes of the early N1 and P2, intermediate N2, or late P3 components.

## Materials and Methods

### Participants

Thirty Chinese college students (16 males; aged between 17 and 24 years, mean age: 19 ± 2.3 years) participated in this study as paid volunteers. *A posteriori* statistical power analysis via G^∗^power was conducted (Faul et al., 2009), and confirmed that our sample size was large enough to obtain high statistical power (observed power = 0.8, at an alpha of 0.05). All had normal or corrected-to-normal vision and were free from any neurological dysfunctions. Informed consent was obtained prior to the study, and payment was given after experiment. The study was approved by the Ethics Committee of Hunan Normal University.

### Materials

The stimuli used in this experiment were 40 colorful pictures depicting the hands of strangers in painful or neutral situations, which have been used in previous studies (Gu and Han, 2007; Fan and Han, 2008). Twenty painful pictures illustrated situations that could happen in everyday life such as a hand trapped in a door or cut by scissors. Each painful picture corresponded to a no-painful picture that showed a similar context, yet without nociceptive component. The mean luminances of painful (19.2 Cd/m^2^) and no-painful (18.76 Cd/m^2^) picture were matched, *t*(38) = 0.024, *p* = 0.88. The stimuli were presented in the center of a gray background of a 21-inch color monitor, all of which was 7 cm × 5.5 cm (width × height), and subtended a visual angle of 4° × 3.15° at a viewing distance of 100 cm.

### Experimental Task and Procedure

**FIGURE 1:**
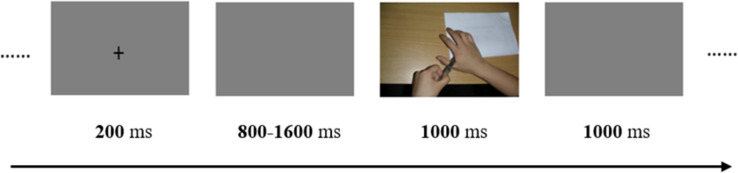
Illustration of the temporal sequence of an individual trial.

Each trial began with a fixation cross at the center of the screen for 200 ms, which was followed by a blank interval that varied randomly from 800 to 1600 ms. Subsequently, the picture was presented lasting for 1000 ms. Participants were instructed to judge each picture as painful or no-painful by their left or right index finger. The assignment of the left or right index finger to the painful and no-painful stimuli was counterbalanced across participants. After the picture disappeared, there was an interval of 1000 ms before the onset of the next trial (see Figure 1). The entire experiment consisted of four blocks of 160 trials (each picture was presented one time in a random order in each block).

After the electroencephalography (EEG) recording session, participants were instructed to evaluate the intensity of pain supposedly felt by the model of each picture as well as the degree of their own unpleasantness when they saw each picture. The evaluations were measured using a 6-point scale (from 1 to 6, representing no pain to extremely painful or no unpleasantness to extremely unpleasant) with the Face Pain Scale-Revised (FPS-R) adapted from the Faces Pain Scale (Bieri et al., 1990), which consisted six facial expressions varying from neutral to extremely painful or unpleasant.

At the end, participants completed the 24-item SCS on a seven-point Likert scale (1 = strongly disagree, 7 = strongly agree) so as to evaluate their interdependence and independence of self-construal (Singelis, 1994). Participants’ self-construal index was calculated by subtracting the sum score of independent items from that of interdependent items (Chiao et al., 2010b). Higher scores reflect a greater degree of interdependence orientation.

### Electrophysiological Recording and Analysis

The EEG was continuously recorded from 64 scalp electrodes mounted on an elastic cap (Brain Products, Germany) in conformity to the extended 10–20 system while being referenced online to the FCz electrode. Vertical and horizontal electro-oculogram (EOG) were recorded from above and below the left eye and at left and right outer canthi, respectively. The gain and dynamic range of the amplifier were 0.1 μV and ±3.28 mV, respectively. The EEG and EOG were amplified with a 0.05–100-Hz bandpass and digitized at a sampling rate of 500 Hz. Impedances of all electrodes were kept below 10 kΩ.

The electrophysiological data was processed offline using the Brain Vision Analyzer 2.1 software (Brain Products, Germany). It was first re-referenced to the average of the left and right mastoid electrode, and then corrected on the VEOG and HEOG based on the independent component analysis (ICA), high-pass filtered above 0.1 Hz and low-pass filtered below 30 Hz, epoched from 200 ms before onset of the stimulus to 1000 ms after its onset, and corrected with a baseline of mean voltage over the 200 ms pre-stimulus interval. Trials of each condition (pain and neutral) without any in which EEG voltages exceeded a threshold of ±80 μV were averaged separately followed by the baseline correction.

Previous ERP studies in the domain of pain empathy showed that both painful and no-painful stimuli elicited obvious N1 component between approximately 90 and 130 ms, P2 component between approximately 140 and 200 ms, N2 component between approximately 200 and 280 ms over the frontal-central regions, and P3 component after 380 ms over the central–parietal regions (Fan and Han, 2008; Han et al., 2008; Decety et al., 2010; Ibáñez et al., 2011). Thus, according to previous studies and the observation on the present ERP waveforms, and in order to investigate the pain × electrode interaction, the average amplitudes at 80–120 ms (N1), 140–190 ms (P2), 200–300 ms (N2), and 300–600 ms (P3) were observed at frontal (F3, Fz, F4), fronto-central (FC3, FCz, FC4), central (C3, Cz, C4), centro-parietal (CP3, CPz, CP4), and parietal (P3, Pz, P4) electrodes. Moreover, in order to investigate the detailed time-course of cognitive processing, the averaged amplitudes in each 100-ms interval of the 300–600-ms time window were measured for the P3 component.

A two-way repeated measures analysis of variance (ANOVA) was conducted on the mean amplitudes of the P2 and N2 components, with the pain (two levels: painful, no-painful) and electrode (five levels: frontal, fronto-central, central, centro-parietal, and parietal regions) as within-subject factors. Moreover, in order to investigate the timing features of the P3 component, we added timing (3 levels: 300–400, 400–500, 500–600 ms) as another factor and thus a three-way repeated-measures ANOVA was conducted on the mean amplitudes of the P3 component. Both accuracy and reaction time were subject to a paired-samples *t*-test. The *p*-values of all analyses were corrected according to Greenhouse–Geisser if violation of sphericity existed. Partial eta-squared ($\eta_{\text{p}}^{2}$) was reported to illustrate the effect size of the analysis of variance (ANOVA). False discovery rate (FDR) correction was applied for multiple comparisons.

## Results

### Behavioral Results

The results showed that response accuracy between painful and no-painful condition did not show significant difference [*t*(29) = −1.94, *p* > 0.05]. Yet participants responded faster to neutral than to painful stimuli [*t*(29) = 6.46, *p* < 0.001]. In addition, both the degree of perceived pain and self-unpleasantness were higher in painful than in no-painful stimulus conditions (*p* < 0.001).

**FIGURE 2:**
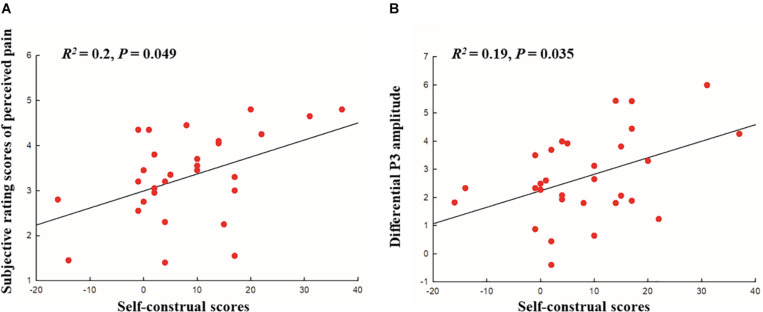
**(A)** Scatter plot with correlation analysis between the self-construal scores (interdependence minus independence) and subjective ratings of perceived pain (painful minus no-painful stimuli), **(B)** Scatter plot with correlation analysis between the self-construal scores (interdependence minus independence) and the differential P300 amplitude at 400–500 ms (painful minus no-painful).

Moreover, we conducted a regression analysis to examine the relationship between the level of interdependent self-construal (interdependence minus independence scores) and rating scores of perceived pain and self-unpleasantness (painful minus no-painful stimulus conditions). The mean score for interdependent self-construal (interdependence minus independence scores) was 8.27 (±11.41 SD). The rating scores of perceived pain for painful and no-painful stimuli were 4.44 (±0.98 SD) and 1.13 (±0.24 SD), respectively. The rating scores of self-unpleasantness for painful and no-painful stimuli were 3.99 (±1.01 SD) and 1.12 (±0.21 SD), respectively. A linear regression revealed a significant effect of the interdependence on perceived pain [*R*^2^ = 0.2, *t*(28) = 2.63, FDR-corrected *p* = 0.049, see Figure 2A], but not on self-unpleasantness [*R*^2^ = 0.07, *t*(28) = 1.49, FDR-corrected *p* = 0.26].

### ERP Results

The ANOVA for mean amplitudes of N1 and P2 showed no significant main effect of pain or interaction effect between pain and electrode (see Table 1).

**TABLE 1:** Summary of results of ANOVAs for the Nl, P2, N2, 300–400, 400–500, and 500–600 ms time windows.

ERP components	Pain		Electrode		Pain × Electrode	
	*F*	*P*	*F*	*P*	*F*	*P*
Nl (80–120 ms)	0.02	0.88	43.97	0.00	1.96	0.17
P2 (140–190 ms)	0.06	0.81	4.12	0.047	0.38	0.63
N2 (200–300 ms)	9.99	0.004	65.09	0.00	10.08	0.00
300–400 ms	59.43	0.00	77.75	0.00	8.16	0.001
400–500 ms	96.09	0.00	36.19	0.00	0.71	0.48
500–600 ms	56.34	0.00	12.01	0.001	2.06	0.15

**FIGURE 3:**
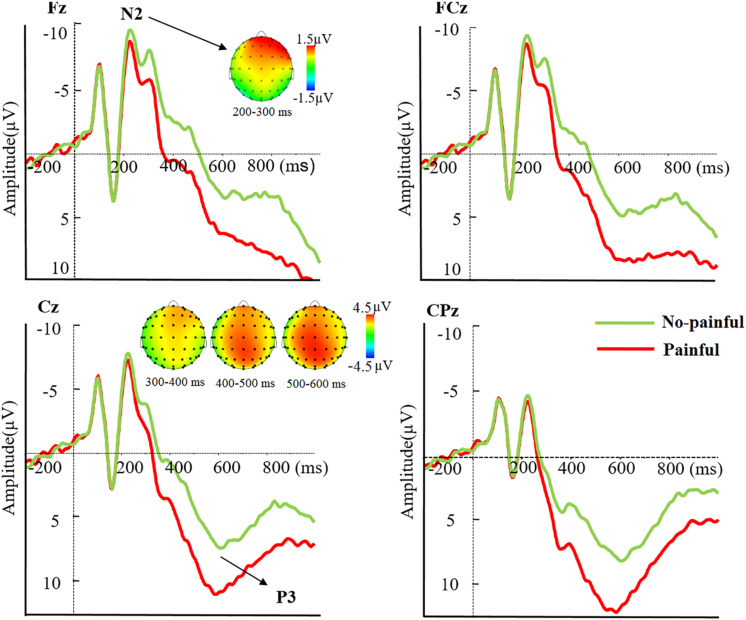
Averaged ERPs at Fz, FCz, Cz, and CPz to painful (red lines) and no-painful (green lines) stimuli, and topographical maps of voltage amplitudes for painful minus no-painful stimuli difference at the N2 and P3 components.

The N2 component during 200–300 ms showed a significant main effect of pain, such that a decreased N2 amplitude was elicited by painful than no-painful stimuli [*F*(1,29) = 9.99, *p* = 0.004, $\eta_{\text{p}}^{2}=0.26$] (see Figure 3). Moreover, the interaction between pain and electrode was significant [*F*(4,116) = 10.08, *p* < 0.001, $\eta_{\text{p}}^{2}=0.26$]. The pain effect, obtained by subtracting the no-painful from painful conditions, was larger over the frontal than other electrodes (*p* < 0.05).

The three-way repeated measures ANOVA on mean P3 amplitudes showed a significant three-way interaction amongst pain, electrode and timing [*F*(8,232) = 11.63, *p* < 0.001, $\eta_{\text{p}}^{2}=0.29$]. In order to disentangle the three-way interaction, we tested significances of main effects of pain and electrode, as well as their interaction effect in each of the three time windows. There was a main effect of pain on mean P3 amplitudes observed during 300–400 ms [*F*(1,29) = 59.43, *p* < 0.001, $\eta_{\text{p}}^{2}=0.67$], 400–500 ms [*F*(1,29) = 96.09, *p* < 0.001, $\eta_{\text{p}}^{2}=0.77$], and 500–600 ms [*F*(1,29) = 56.34, *p* < 0.001, $\eta_{\text{p}}^{2}=0.66$] (see Figure 3), with larger P3 amplitudes elicited by painful than no-painful stimuli. Moreover, a significant interaction effect between pain and electrode was observed during 300–400 ms [*F*(4,116) = 8.16, *p* = 0.001, $\eta_{\text{p}}^{2}=0.22$], with larger pain effect, obtained by subtracting the no-painful from painful conditions, observed over frontal and fronto-central than other electrodes (*p* < 0.05). However, no such interaction effects were observed during 400–500 and 500–600 ms (see Table 1).

In order to examine whether the level of dispositional self-construal could modulate the neural empathic responses to pain, we conducted a regression analysis, with the level of interdependent self-construal (interdependence minus independence scores) as the independent variable, and the N2 and P3 amplitude differences (painful minus no-painful stimulus conditions) as the dependent variables. As the pain effects were more obvious over frontal electrodes during the N2 period and 300–400 ms, mean amplitude differences averaged across these electrodes were used for the regression analysis. As for the 400–500- and 500–600-ms intervals, mean amplitude differences averaged across all electrodes were used because there were no significant pain by electrode interactions effect observed during these time intervals. The linear regression revealed a significant effect of the interdependence on the amplitude difference of P3 at 400–500 ms [*R*^2^ = 0.19, *t*(28) = 2.6, FDR-corrected *p* = 0.035, see Figure 2B], but not on the amplitude differences of N2 [*R*^2^ = 0.04, *t*(28) = −1.12, FDR-corrected *p* = 0.32] and P3 at 300–400 ms [*R*^2^ = 0.03, *t*(28) = 0.93, FDR-corrected *p* = 0.36] and 500–600 ms [*R*^2^ = 0.05, *t*(28) = 1.2, FDR-corrected *p* = 0.34].

## Discussion

The present study examined the modulation effect of the dispositional self-construal on neural empathic responses to pain and further determined whether the modulation effect occurred during the early automatic emotional sharing stage, the later attention orienting stage, or the late cognitive evaluation stage. The findings showed that painful stimuli elicited decreased N2 amplitudes and larger P3 amplitudes than those by no-painful stimuli. Moreover, the level of interdependent self-construal positively correlated with the amplitude difference of P3, but not the N2. The behavioral data also showed that the level of interdependent self-construal could positively predict behavioral ratings of perceived pain, but not self-unpleasantness. These findings suggested that the modulation effect of the dispositional self-construal on neural empathic responses to others’ pain occurred at the later cognitive evaluation stage indexed by the P3 component.

The present study did not show differences (pain vs. non-painful) for the early N1 and P2 components, which was inconsistent with previous studies showing significant main effects of pain on the N1 or P2 components (Fan and Han, 2008; Decety et al., 2010). These inconsistencies might be due to the fact that the present study used different experimental materials and tasks from these studies. For example, the pain-related stimuli used in the present study were pictures depicting the hands of strangers in painful situations, whereas the pictures of faces in painful situations were used in Decety et al.’s (2010) study, and both pictures and cartoons of hand stimuli were used in Fan and Han’s (2008) study. In addition, the task used in the present study was a pain judgment task, whereas both pain judgment and counting tasks were used in Fan and Han’s (2008) study. Moreover, a recent meta-analysis research suggested that the early N1 component are not reliably associated with the observation of pain in others (Coll, 2018).

The N2 component was considered a “semiautomatic” component, reflecting the final stage of automatic attention-related neural mechanisms (Daffner et al., 2000). More specifically, the N2 was thought to reflect the attention orienting response to emotionally salient and biologically important stimuli (Nagy et al., 2003; Carretié et al., 2004). Thus, the N2 effect observed in the present study may reflect the attention orienting response to pain-related stimuli as the perception of pain is significant for survival and adaptation. Our results were consistent with previous studies showing that painful stimuli elicited decreased ERPs during 200–280 ms (N240) at the anterior frontal electrodes as compared to no-painful stimuli, and this pain effect was evident in both pain judgment and counting tasks (Fan and Han, 2008). Moreover, Jiang et al. (2014) also found that painful stimuli, as compared to no-painful stimuli, elicited decreased ERP waves during 232–332 ms named the N2 and N320 components (Jiang et al., 2014). However, the modulation effect of the dispositional self-construal on neural empathic responses to pain did not occur in this processing stage.

We also observed larger P3 amplitudes elicited by painful than no-painful stimuli. It has been widely accepted that the P3, a positive shift of ERP wave that appears approximately 300 ms after the stimulus onset, reflects multiple cognitive processes, including top-down controlled attentional processes, cognitive evaluation of stimulus meaning, and the updating of representations in working memory (Donchin and Coles, 1988; Ito et al., 1998; Polich, 2007). Pascalis et al. (1999) found that the more painful one felt, a larger P3 peak should be elicited (Pascalis et al., 1999). Pain is a warning mechanism of an organism and signals a potential threat in the environment (Williams, 2002). Thus, the larger P3 amplitudes for painful stimuli may reflect increased cognitive evaluations and attentional resources toward pain-related information. Our results were also consistent with previous ERP studies showing increased P3 amplitudes for painful than for no-painful stimuli, which reflected a later controlled process of empathy for pain (Fan and Han, 2008; Decety et al., 2010; Meng et al., 2012).

More importantly, we found a linear relationship between the level of dispositional self-construal and amplitude difference of P3; the higher their level of the interdependent self-construal, the stronger their neural empathic responses to painful stimuli. Moreover, the level of interdependent self-construal could also positively predict behavioral ratings of perceived pain, but not self-unpleasantness. It has been suggested that empathy is an outcome of emotion identification or affect sharing processes, and these two processes are independent (Coll et al., 2017). In the present study, the rating of other’s pain actually represents the emotion identification process, whereas the rating of self-unpleasantness represents the affect sharing process. Thus, the present behavioral and ERP findings both indicated that the interdependent self-construal could modulate the cognitive-evaluative component of empathy rather than the affective component. On the other hand, these findings also provide some support for Coll et al.’ (2017) view that emotion identification and affect sharing processes in pain empathy are independent.

Markus and Kitayama (1991) argued that individuals with high interdependent self-construal might be especially likely to pay attention to others and the social context of interaction, and thus resulted in elaborate cognitive representations of others and self-representations that include social contexts. Previous fMRI study found that, as compared to the general self-judgment, individuals with dispositional interdependent self-construal or primed with interdependent self-construal both showed increased activation in medial prefrontal cortex (MPFC) for the contextual self-judgment, which highlights self-representation in a social context (Chiao et al., 2010a,b). Moreover, the level of interdependent self-construal (interdependent minus independent) positively correlated with the MPFC responses to contextual self-judgments (contextual minus general) (Chiao et al., 2010b). Similarly, van Horen et al. (2008) also found that individuals with interdependent self-construal attached more importance to social goals (e.g., in harmony with others) compared to individual goals (e.g., to be ambitious) (van Horen et al., 2008). Thus, consistent with these studies, the focus on the interconnectedness and interdependence with others in individuals with higher interdependent self-construal might also contribute to enhanced behavioral and neural empathic responses to others’ pain. In addition, it has been suggested that individuals with interdependent self-construal are associated with prosocial motivation and affiliative tendencies such that individuals primed with interdependent self-construal exhibited a higher level of cooperation than those primed with independent self-construal in a social dilemma task (Utz, 2004). Moreover, Niiya and Crocker (2019) found that the indices of interdependence showed moderately positive correlations with compassionate goals. Thus, the higher sensitivity to others’ pain observed in individuals with higher interdependent self-construal might be associated with their high level of prosocial motivation, affiliative tendencies, or compassion.

In addition, some limitations of the present work should be noted. First, all participants who attended the present study are East Asians, whose interdependency is dominant (Markus and Kitayama, 1991). Thus, it remains unknown whether the modulation effect of dispositional self-construal on the neural empathic responses to others’ pain exists in the Westerners with a dominant independent self-construal. Second, the pain-related stimuli used in the present were pictures depicting the hands of strangers in painful situations. It is still unknown whether and how dispositional self-construal could modulate neural responses to faces with pain expressions or in painful situations, especially to the suffering faces of ingroup and outgroup members. These questions should be addressed in future studies.

Taken together, in accordance with previous studies demonstrating the modulation effect of temporary shifts in self-construal on the neural empathic responses to others’ pain (Jiang et al., 2014), the present study also provides evidence that the dispositional self-construal could also modulate the neural empathic responses to others’ pain. However, Jiang et al. (2014) found that temporary shifts in self-construal only modulated the ERP component of empathy indexed by the N2 and N320 components, but not the late P3 component. By contrast, the present study found that the modulation effect of dispositional self-construal on neural empathic responses occurred at the late P3 stage, but not the N2 stage. These findings indicated that the temporary shifts in self-construal and dispositional self-construal might play a different role in the neural empathic responses to others’ pain. However, it remains unknown whether the influences of temporary and dispositional self-construal on perception of others’ pain recruit same or distinct neural substrates. Future studies should unravel the neural substrates that mediate these modulation effects using high-spatial-resolution fMRI.

## Data Availability Statement

The datasets generated for this study are available on request to the corresponding author.

## Ethics Statement

The studies involving human participants were reviewed and approved by the Ethics Committee of Hunan Normal University. The patients/participants provided their written informed consent to participate in this study.

## Author Contributions

JC: study design, data analysis, and manuscript writing. BC: data analysis and manuscript writing. WL and YS: manuscript writing. LL, HS, and RW: data analysis. All authors contributed to the article and approved the submitted version.

## Conflict of Interest

The authors declare that the research was conducted in the absence of any commercial or financial relationships that could be construed as a potential conflict of interest.

## References

[B1] Bieri D. Reeve R. A. Champion G. D. Addicoat L. Ziegler J. B. (1990). The faces pain scale for the self-assessment of the severity of pain experienced by children: development, initial validation, and preliminary investigation for ratio scale properties. *Pain* 41 139–150. 10.1016/0304-3959(90)90018-9 2367140

[B2] Carretié L. Hinojosa J. A. Martín-Loeches M. Mercado F. Tapia M. (2004). Automatic attention to emotional stimuli: neural correlates. *Hum. Brain Mapp.* 22 290–299. 10.1002/hbm.20037 15202107 PMC6871850

[B3] Chiao J. Y. Harada T. Komeda H. Li Z. Mano Y. Sadato N. (2010a). Dynamic cultural influences on neural representations of the self. *J. Cogn. Neurosci.* 22 1–11. 10.1162/jocn.2009.21192 19199421

[B4] Chiao J. Y. Harada T. Komeda H. Li Z. Mano Y. Sadato N. (2010b). Neural basis of individualistic and collectivistic views of self. *Hum. Brain Mapp.* 30 2813–2820. 10.1002/hbm.20707 19107754 PMC6870804

[B5] Chiao J. Y. Mathur V. A. Harada T. Lipke T. (2009). Neural Basis of Preference for Human Social Hierarchy versus Egalitarianism. *Ann. N.Y. Acad. Sci.* 1167 174–181. 10.1111/j.1749-6632.2009.04508.x 19580563

[B6] Coll M. P. (2018). Meta-analysis of ERP investigations of pain empathy underlines methodological issues in ERP research. *Soc. Cogn. Affect. Neurosci.* 13 1003–1017. 10.1093/scan/nsy072 30137502 PMC6204484

[B7] Coll M.-P. Viding E. Rütgen M. Silani G. Lamm C. Catmur C. (2017). Are we really measuring empathy? Proposal for a new measurement framework. *Neurosci. Biobehav. Rev.* 83 132–139. 10.1016/j.neubiorev.2017.10.009 29032087

[B8] Cross S. E. Hardin E. E. Gercek-Swing B. (2011). The What, How, Why, and Where of Self-Construal. *Pers. Soc. Psychol. Rev.* 15 142–179. 10.1177/1088868310373752 20716643

[B9] Cui F. Ma N. Luo Y. (2016). Moral judgment modulates neural responses to the perception of other’s pain: an ERP study. *Sci. Rep.* 6:20851. 10.1038/srep20851 26865250 PMC4749990

[B10] Daffner K. R. Mesulam M. M. Scinto L. F. M. Calvo V. Faust R. Holcomb P. J. (2000). An electrophysiological index of stimulus unfamiliarity. *Psychophysiology* 37 737–747. 10.1111/1469-8986.3760737 11117454

[B11] Decety J. Jackson P. L. (2004). The functional architecture of human empathy. *Behav. Cogn. Neurosci. Rev.* 3:71. 10.1177/1534582304267187 15537986

[B12] Decety J. Yang C. Cheng Y. (2010). Physicians down-regulate their pain empathy response: an event-related brain potential study. *Neuroimage* 50 1676–1682. 10.1016/j.neuroimage.2010.01.025 20080194

[B13] Donchin E. Coles M. G. (1988). Is the P300 component a manifestation of context updating? *Behav. Brain Sci.* 11 357–374. 10.1017/S0140525X00058027

[B14] Fan Y. Han S. (2008). Temporal dynamic of neural mechanisms involved in empathy for pain: an event-related brain potential study. *Neuropsychologia* 46 160–173. 10.1016/j.neuropsychologia.2007.07.023 17825852

[B15] Faul F. Erdfelder E. Buchner A. Lang A.-G. (2009). Statistical power analyses using G^∗^ power 3.1: tests for correlation and regression analyses. *Behav. Res. Methods* 41 1149–1160. 10.3758/BRM.41.4.1149 19897823

[B16] Gu X. Han S. (2007). Attention and reality constraints on the neural processes of empathy for pain. *Neuroimage* 36 256–267. 10.1016/j.neuroimage.2007.02.025 17400480

[B17] Han S. Fan Y. Mao L. (2008). Gender difference in empathy for pain: an electrophysiological investigation. *Brain Res.* 1196 85–93. 10.1016/j.brainres.2007.12.062 18221733

[B18] Harada T. Li Z. Chiao J. Y. (2010). Differential dorsal and ventral medial prefrontal representations of the implicit self-modulated by individualism and collectivism: an fMRI study. *Soc. Neurosci.* 5 257–2571. 10.1080/17470910903374895 20178036

[B19] Ibáñez A. Hurtado E. Lobos A. Escobar J. Trujillo N. Baez S. (2011). Subliminal presentation of other faces (but not own face) primes behavioral and evoked cortical processing of empathy for pain. *Brain Res.* 1398 72–85. 10.1016/j.brainres.2011.05.014 21624566

[B20] Ito T. A. Larsen J. T. Smith N. K. Cacioppo J. T. (1998). Negative information weighs more heavily on the brain: the negativity bias in evaluative categorizations. *J. Pers. Soc. Psychol.* 75:887. 10.1037/0022-3514.75.4.887 9825526

[B21] Jiang C. Varnum M. E. W. Hou Y. Han S. (2014). Distinct effects of self-construal priming on empathic neural responses in Chinese and Westerners. *Soc. Neurosci.* 9 130–138. 10.1080/17470919.2013.867899 24341541

[B22] Lin Z. Han S. (2009). Self-construal priming modulates the scope of visual attention. *Q. J. Exp. Psychol. Section A* 62 802–813. 10.1080/17470210802271650 18720280

[B23] Liu S. S. Morris M. W. Talhelm T. Yang Q. (2019). Ingroup vigilance in collectivistic cultures. *Proc. Natl. Acad. Sci. U.S.A.* 116 14538–14546. 10.1073/pnas.1817588116 31249140 PMC6642384

[B24] Markus H. R. Kitayama S. (1991). Culture and the self: implications for cognition. emotion, and motivation. *Psychol. Rev.* 98 224–253. 10.1037/0033-295X.98.2.224

[B25] Mathur V. A. Harada T. Lipke T. Chiao J. Y. (2010). Neural basis of extraordinary empathy and altruistic motivation. *Neuroimage* 51 1468–1475. 10.1016/j.neuroimage.2010.03.025 20302945

[B26] Mella N. Studer J. Gilet A. Labouvievief G. (2012). Empathy for pain from adolescence through adulthood: an event-related brain potential study. *Front. Psychol.* 3:501. 10.3389/fpsyg.2012.00501 23189065 PMC3505871

[B27] Meng J. Hu L. Shen L. Yang Z. Chen H. Huang X. (2012). Emotional primes modulate the responses to others’ pain: an erp study. *Exp. Brain Res.* 220 277–286. 10.1007/s00221-012-3136-2 22695721

[B28] Nagy E. Potts G. F. Loveland K. A. (2003). Sex-related ERP differences in deviance detection. *Int. J. Psychophysiol.* 48 285–292. 10.1016/S0167-8760(03)00042-4 12798988

[B29] Niiya Y. Crocker J. (2019). Interdependent = compassionate? Compassionate and self-image goals and their relationships with interdependence in the United States and Japan. *Front. Psychol.* 10:192. 10.3389/fpsyg.2019.00192 30792678 PMC6374305

[B30] Obhi S. S. Hogeveen J. Pascual-Leone A. (2011). Resonating with others: the effects of self-construal type on motor cortical output. *J. Neurosci.* 31 14531–14535. 10.1523/JNEUROSCI.3186-11.2011 21994369 PMC6703414

[B31] Oyserman D. Coon H. M. Kemmelmeier M. (2002). Rethinking individualism and collectivism: evaluation of theoretical assumptions and meta-analysis. *Psychol. Bull.* 128 3–72. 10.1037/0033-2909.128.1.3 11843547

[B32] Pascalis V. D. Magurano M. R. Bellusci A. (1999). Pain perception, somatosensory event-related potentials and skin conductance responses to painful stimuli in high, mid, and low hypnotizable subjects: effects of differential pain reduction strategies. *Pain* 83 499–508. 10.1016/S0304-3959(99)00157-8 10568858

[B33] Polich J. (2007). Updating P300: an integrative theory of P3a and P3b. *Clin. Neurophysiol.* 118 2128–2148. 10.1016/j.clinph.2007.04.019 17573239 PMC2715154

[B34] Sessa P. Meconi F. Castelli L. Dell’Acqua R. (2014a). Taking one’s time in feeling other-race pain: an event-related potential investigation on the time-course of cross-racial empathy. *Soc. Cogn. Affect. Neurosci.* 9:454. 10.1093/scan/nst003 23314008 PMC3989124

[B35] Sessa P. Meconi F. Han S. (2014b). Double dissociation of neural responses supporting perceptual and cognitive components of social cognition: evidence from processing of others’ pain. *Sci. Rep.* 4:7424. 10.1038/srep07424 25502570 PMC4262888

[B36] Singelis T. M. (1994). The Measurement of Independent and Interdependent Self-Construals. *Pers. Soc. Psychol. Bull.* 20 580–591. 10.1177/0146167294205014

[B37] Sui J. Han S. (2007). Self-construal priming modulates neural substrates of self-awareness. *Psychol. Sci.* 18 861–866. 10.1111/j.1467-9280.2007.01992.x 17894602

[B38] Sui J. Hong Y. Liu C. H. Humphreys G. W. Han S. (2013). Dynamic cultural modulation of neural responses to one’s own and friend’s faces. *Soc. Cogn. Affect. Neurosci.* 8 326–332. 10.1093/scan/nss001 22258798 PMC3594724

[B39] Utz S. (2004). Self-construal and cooperation: is the interdependent self more cooperative than the independent self? *Self Identity* 3 177–190. 10.1080/13576500444000001

[B40] van Horen F. Pöhlmann C. Koeppen K. Hannover B. (2008). Importance of personal goals in people with independent and interdependent selves. *Soc. Psychol.* 39 213–221. 10.1027/1864-9335.39.4.213

[B41] Wang C. Ma Y. Han S. (2014). Self-construal priming modulates pain perception: event-related potential evidence. *Cogn. Neurosci.* 5 3–9. 10.1080/17588928.2013.797388 24168205

[B42] Wang C. Wu B. Liu Y. Wu X. Han S. (2015). Challenging emotional prejudice by changing self-concept: priming independent self-construal reduces racial in-group bias in neural responses to other’s pain. *Soc. Cogn. Affect. Neurosci.* 10 1195–1201. 10.1093/scan/nsv005 25605968 PMC4560940

[B43] Williams A. C. D. C. (2002). Facial expression of pain: an evolutionary account. *Behav. Brain Sci.* 25 439–455. 10.1017/S0140525X02000080 12879700

[B44] Xu X. Zuo X. Wang X. Han S. (2009). Do you feel my pain? Racial group membership modulates empathic neural responses. *J. Neurosci.* 29 8525–8529. 10.1523/JNEUROSCI.2418-09.2009 19571143 PMC6665679

